# Recovery of health-related quality of life in ICU patients: a 5-year prospective cohort study

**DOI:** 10.1186/cc14645

**Published:** 2015-03-16

**Authors:** J Hofhuis, HF Van Stel, AJ Schrijvers, JH Rommes, PE Spronk

**Affiliations:** 1 Gelre Hospitals, Apeldoorn, the Netherlands; 2 University Medical Center, Utrecht, the Netherlands

## Introduction

Severe critical illness requiring treatment in the ICU may have a serious impact on patients and their families. However, optimal follow-up periods are not defined and data on health-related quality of life (HRQOL) before ICU admission as well as those beyond 2-year follow-up are limited. The aim of our study was to assess the impact of ICU stay up to 5 years after ICU discharge.

## Methods

We performed a long-term prospective cohort study in patients admitted >48 hours to a medical-surgical ICU. The Short-Form 36 was used to evaluate HRQOL before admission (by proxy within 48 hours after admission of the patient), at ICU discharge and at 1, 2 and 5 years following ICU discharge (all by patients). Changes in HRQOL were assessed using linear mixed modeling.

## Results

We included a total of 749 patients (from 2000 to 2007). At 5 years after ICU discharge, 234 patients could be evaluated. After correction for natural decline in HRQOL, the mean scores of four dimensions - physical functioning (*P *< 0.001), physical role (*P *< 0.001), general health (*P *< 0.001) and social functioning (*P *= 0.003) - were still significantly lower 5 years after ICU discharge compared with their preadmission levels, although effect sizes were small (<0.5).

## Conclusion

Five years after ICU discharge, survivors still perceived a significantly lower HRQOL than their preadmission HRQOL (by proxies), and that of an age-matched general population. Importantly however, after correction for natural decline, the effect sizes were small suggesting that patients regain their age-specific HRQOL 5 years after their ICU stay.

